# Long-Term Environmental Correlates of Invasion by *Lantana camara* (Verbenaceae) in a Seasonally Dry Tropical Forest

**DOI:** 10.1371/journal.pone.0076995

**Published:** 2013-10-22

**Authors:** Geetha Ramaswami, Raman Sukumar

**Affiliations:** 1 Centre for Ecological Sciences, Indian Institute of Science, Bangalore, India; 2 Divecha Centre for Climate Change, Indian Institute of Science, Bangalore, India; University of Waikato (National Institute of Water and Atmospheric Research), New Zealand

## Abstract

Invasive species, local plant communities and invaded ecosystems change over space and time. Quantifying this change may lead to a better understanding of the ecology and the effective management of invasive species. We used data on density of the highly invasive shrub *Lantana camara* (lantana) for the period 1990–2008 from a 50 ha permanent plot in a seasonally dry tropical forest of Mudumalai in southern India. We used a cumulative link mixed-effects regression approach to model the transition of lantana from one qualitative density state to another as a function of biotic factors such as indicators of competition from local species (lantana itself, perennial grasses, invasive *Chromolaena odorata*, the native shrub *Helicteres isora* and basal area of native trees) and abiotic factors such as fire frequency, inter-annual variability of rainfall and relative soil moisture. The density of lantana increased substantially during the study period. Lantana density was negatively associated with the density of *H. isora*, positively associated with basal area of native trees, but not affected by the presence of grasses or other invasive species. In the absence of fire, lantana density increased with increasing rainfall. When fires occurred, transitions to higher densities occurred at low rainfall values. In drier regions, lantana changed from low to high density as rainfall increased while in wetter regions of the plot, lantana persisted in the dense category irrespective of rainfall. Lantana seems to effectively utilize resources distributed in space and time to its advantage, thus outcompeting local species and maintaining a population that is not yet self-limiting. High-risk areas and years could potentially be identified based on inferences from this study for facilitating management of lantana in tropical dry forests.

## Introduction

The success of invasive species is generally attributed to the properties of the invading species [1], [2], those of the invaded communities and environment [3], [4] or propagule pressure [5], [6], with recent emphasis on the synthesis of these theories [7], [8]. However, the dynamics of invasive species – their population structure, impacts and interactions with environmental variables such as weather and disturbances, change with time [9]. Often invasive plants are known to persist benignly in low numbers for long periods of time before exhibiting sudden changes such as local population explosion or range expansion which could be associated with either changes in the invading species itself or in the abiotic environment or biotic interactions [10]. One way to understand these processes is to monitor populations over sufficiently long time periods. Although the need for long-term monitoring has been emphasised as a requirement for understanding invasive species dynamics and changing impacts [11], only a small proportion of studies track invasive plants over long time periods [9]. Understanding the factors that affect the populations of invasive species over time could have implications for the management and restoration of invaded ecosystems. At small spatial scales, it is possible to have detailed long-term data about the demography of invasive species (e.g. [12]) as well as their interactions with the abiotic environment (e.g. [13]).

*Lantana camara* L. (henceforth lantana) is a thicket-forming woody shrub of tropical American origin and is currently a widespread invasive, occurring widely across the tropical and subtropical regions [14]. Seasonally dry tropical forests (250–2000 mm annual rainfall in 4–6 months of the year), though comprising 42% of tropical ecosystems of the world, are relatively understudied compared to wetter tropical forests [15], [16]. The aim of this study was to understand what spatially and temporally variable abiotic and biotic factors influenced invasion by lantana, defined as its transition from a qualitatively determined lower density category to a higher one, using a time-series data set at a fine spatial scale.

We expected the following biotic factors to influence the spread of lantana -

1. Competition – Resident species, competing for limiting resources, have been cited as a probable mechanism for resistance to invasion [17], [18]. In the absence of disturbances, surplus resources that can be utilized by invading species would become unavailable. Thus, competition from resident species could possibly result in the suppression of invading species. Alternately, invasive species could persist through such ‘lean periods’ at lower density and expand opportunistically under future favourable conditions [19]. The local species pool could comprise of native as well as other introduced species. We hypothesise that with increasing density of competing resident shrubs and grasses, lantana would tend to persist at low densities or go from higher to lower density categories. Once higher lantana densities are attained, it was expected that there would be density-dependent limiting of invasion. We thus expected intra-specific competition to reduce lantana invasion.
2. Basal area of trees – The presence of trees has been shown to decrease the rate of spread of shrubby invasives [13]. Invasive species like lantana have been shown to perform better in forest edges and gaps than in forest interiors, with percent cover and relative growth rate decreasing with increasing distance from the centre of a gap [20], [21]. Larger gaps have also been shown to support larger sub-populations of lantana and larger individuals [20]. We expected higher basal area of trees to be associated with greater canopy cover and thus lower light availability at ground level. We thus hypothesise that with increasing basal area of trees in space and time, the rate of lantana invasion would be low.

We expected the following abiotic factors to influence the intensity of lantana invasion -

1. Fire – It has been speculated that disturbances create spurts in unused resources in a habitat that can be utilized by invading species [3]. Fire is one such disturbance and has been implicated in the spread and persistence of invasive species [22]. Invasive plants that are adapted to coppicing post-fire, rapidly gain biomass and thus become fuel for further fires, creating a positive feedback cycle resulting in their persistence. A similar framework has also been hypothesised as a mechanism for spread of lantana [23]. Although the role of fire on the spread of shrubby invasive species in general and vice-versa is relatively poorly understood [24], we nevertheless hypothesise that the occurrence of fire facilitates lantana invasion over long time scales.
2. Spatial and temporal availability of moisture – While the availability of surplus resources has been shown to support greater invasive abundance and diversity, invasive species have also been hypothesised to grow and persist better than native species under resource-poor conditions by virtue of having traits that maximise the efficiency of utilisation of available resources [25]. Topographic heterogeneity and seasonality or inter-annual variability in rainfall respectively create resource (i.e. moisture) poor conditions in space and time, respectively. Invasive species may have physiological traits that make them more tolerant to water-stress conditions as compared to native species [26]. *Lantana camara* has been shown to have drought-avoidance strategies – a deep root system and high water retention – thus avoiding damage to photosynthetic apparatus even in the driest regions of arid islands of the Galapagos [27]. This is different from the shallow rooted drought-tolerance strategies of a native congener. In spite of such physiological strategies, however, growth rates of lantana were lower in drier compared to wetter regions of the islands [27]. Soil moisture, affected by local topography, could thus influence invasion by exotic plants. Another example is the invasive herb *Alliaria petiolata* (Bieb.) Cavara & Grande, which had higher productivity and survival in high soil-moisture stream-bottom sites than in upland regions with drier soils [28]. We thus hypothesise that lantana invasion would occur more frequently in moister areas.

We used a cumulative link mixed-effects regression approach at a small but fine spatial scale using 18 years (1990–2008) of data from a permanent 50 ha tropical dry forest plot in southern India. We used indicators of competition, disturbance and available resources at a given time step to understand transition of lantana in a given grid cell from one density class to another, in the next time step. The model so developed helps us understand not only the individual influence of each factor but also interactions between factors on lantana invasion.

## Materials and Methods

### Study Site

**Figure 1:**
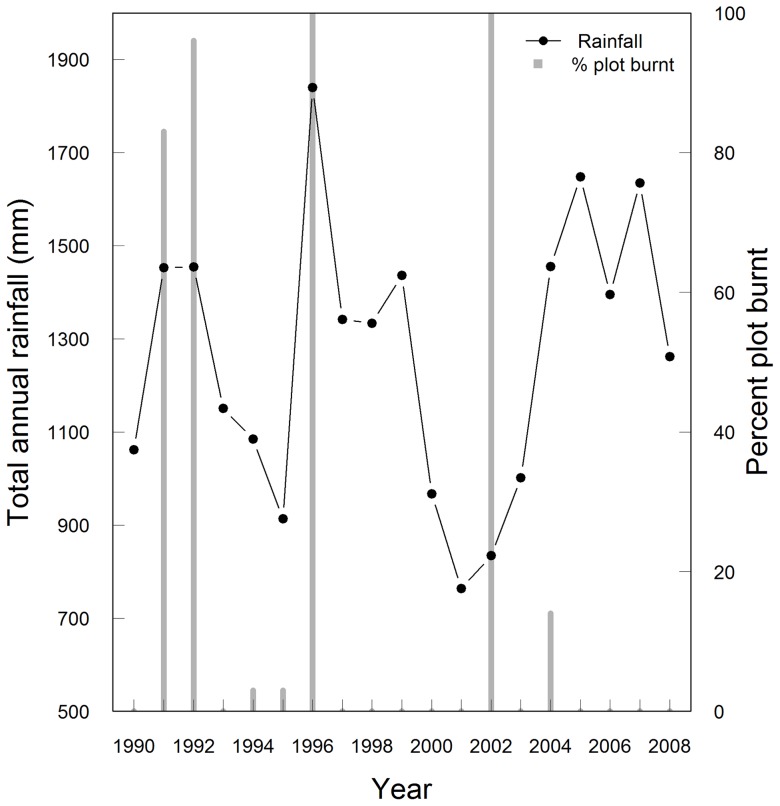
Interannual variability in rainfall and fire at the Mudumalai Forest Dynamics Plot. The solid line and dots represent total annual rainfall (mm) while the grey bars represent the percentage of 10 m×10 m plots burnt each year. It must be noted that fires usually occur during the dry season of each year and are not related to the total rainfall in that year.

The 50 ha Mudumalai Forest Dynamics Plot (MFDP) is located centrally in the tropical dry forests of Mudumalai (11°30′– 11°39′ N, 76°27′–76°43′E; 321 km^2^) of southern India [29]. The MFDP received 1265±292 mm rainfall annually for the period 1990–2008. Partial as well as plot-wide ground fires have occurred in the MFDP in 1991, 1992, 1994, 1996, and 2002 (Fig. 1) during this period. The forest is characterised by an open canopy supporting the persistence of ‘ground vegetation’ that includes grasses such as *Themeda cymbaria* Hack. and *Cymbopogon flexuosus* (Steudel) Watson, ephemeral herbs, as well as the alien invasive species lantana and *Chromolaena odorata* (L.) King & Robinson. Although lantana infestations have been reported in the Gudalur area (15 km from the MFDP) from the early 1900s [30] and has been observed elsewhere in Mudumalai since the late 1970s (R.S. pers. obs.), its presence at high abundance in the MFDP seems fairly recent.

### Data

The 1 km ×0.5 km MFDP is gridded into 20 m×20 m plots, each of which is further sub-divided into four 10 m×10 m sub-plots (our unit of measurement and henceforth referred to as ‘cell’) arranged in 50 rows and 100 columns. Since 1989 an observer (mostly C.M. Bharanaiah) has been visually estimating and assigning the ground vegetation groups – grasses, bamboo, ephemerals, *C. odorata* and lantana – to one of five qualitative density categories – absent, just present, common, dense, very dense (see Figure S1.1 in Appendix S1 for an example). Along with data on ground vegetation, the cells that were burnt during the dry months preceding the enumeration are also recorded. In the years 1992, 1996 and 2002, plot-wide fires had burnt all ground vegetation and data were thus not collected for these years. Excepting 1995 and 1998, the ground vegetation data are available for all years starting from 1989 to present. Permissions for field work at this site were granted by the Tamil Nadu Forest Department (Tamil Nadu state, India).

### Characterizing Biotic Factors

Intraspecific and interspecific competition from ground vegetation was quantified based on the biomass of three ground vegetation groups namely – lantana, grasses and *C. odorata*. In August 2011, ten 1 m ×1 m plots corresponding to the four qualitative density classes – ‘present’, ‘common’, ‘dense’ and ‘very dense’ of lantana, grasses and *C. odorata* – were sampled outside the MFDP (n = 10 plots ×4 density categories ×3 vegetation groups  = 120 sampling plots). The vegetation within each sample plot was harvested, dried and weighed. An average of the dry wt m^−2^ was used as an estimate of the biomass for each density class of each vegetation group. In this analysis, the ‘absent’ and the ‘present’ categories of lantana were combined into a single density class ‘present’ as these categories showed high spatial variability and could not be reliably distinguished from one another. The ‘common’ and ‘dense’ categories were also combined as the former category occurred very rarely and had biomass roughly comparable to the latter category (Table 1). The higher density categories represent established breeding plants that contribute to local intensification of invasion. We thus used three categories of lantana abundance – present, common and very dense – for this analysis. Within each vegetation group, the ‘present’ category was used as a reference and the ratio between this category and all other categories were used as scores for each density class (Table 1). To assess intraspecific competition, density scores for lantana based on biomass values were assigned to each cell for each year. To assess the influence of heterospecific competition on lantana spread, density scores of tall grasses, the shrub *C. odorata* and stem densities of the understorey shrub *Helicteres isora* L. were considered. The sum of density scores of tall grasses and *C. odorata* in a focal cell was used as a measure of heterospecific competition. Competition by *H. isora* was quantified as the sum of stems >1 cm dbh in a focal cell. We used the total basal area of woody stems >1 cm dbh as a measure of competition from other woody plants, as well as a proxy for shading at ground level.

**Table 1:** Estimated mean biomass (in g m^−2^, ±1 standard deviation) of three vegetation categories measured in the Mudumalai Forest Dynamics Plot.

Ground vegetation	Density category	Biomass	± SD	Density score
*Lantana camara*	Absent	0		0
	Present	48.3	±15.6	1
	Common	503.1	±30.5	10
	Dense	858.5	±154.2	18
	Very dense	2038.5	±226.0	42
*Chromolaena odorata*	Absent	0		0
	Present	49.8	±11.4	1
	Common	118	±75.9	2
	Dense	728.5	±261.0	15
	Very dense	1165.9	±348.2	23
Grasses	Absent	0		0
	Present	95.5	±27.1	1
	Common	227.6	±73.8	2
	Dense	704.2	±187.5	7
	Very dense	813.2	±170.5	9

Density scores have been assigned to each category as the ratio between that category and the ‘present’ category.

### Characterizing Abiotic Factors

In order to determine the influence of interannual variability in rainfall on lantana spread, we used yearly data from a weather station maintained by us at Kargudi, about 3 km from the MFDP. We assumed that all cells in the plot received the same amount of rain, and rainfall was therefore held constant across the plot. Since enumeration began in June of a year and extended up to December, the rainfall received between June of one year to May of the following year was considered as annual rainfall received between two consecutive enumerations. For years when plot-wide fires had occurred (1992, 1996, 2002), or when ground vegetation data were not available (1995, 1998), rainfall was averaged for the duration between two valid enumerations. To understand the effects of spatial availability of moisture on change in lantana density in the plot, we used a topography-based static measure of soil moisture – the topographic wetness index, henceforth referred to as TWI [31] (Beven and Kirkby 1979). The TWI values for each cell were derived from a digital elevation map with a resolution of 10 m×10 m from a catchment area of 20 km^2^ centred around the MFDP using the software SAGA version 2.0.5 (System for Automated Geoscientific Analyses, http://www.saga-gis.org). In the MFDP, TWI values ranged from a minimum of 6.8 in the driest part of the plot to a maximum of 13.1 near streams. Since TWI is based only on the relative elevation of a cell with respect to surrounding cells, this measure remained static in time. Cell-wise information on the occurrence of fire was used to determine the influence of fire on lantana invasion. When a cell experienced consecutive fires between two time steps, the fire instances were summed. We did not have a direct measure of overall light availability at ground level for the duration of this study. However, an examination of monthly averages of light availability measured at Kargudi for the years 2005 to 2008, revealed that higher rainfall was correlated with low light availability (Spearman's rho  = −0.6, Figure S1.2 in Appendix S1). We thus assumed that higher rainfall corresponded to lower global solar insolation at the plot. We also assumed that greater the area occupied by other trees in a cell, greater is the shading at ground level. Since shading at ground level was likely to be correlated with rainfall, we did not include any proxies for the same in our model.

### Statistical Analyses

The density states of lantana increased in an ordinal fashion. We therefore chose a proportional odds logistic regression to assess the changes in lantana density. Since this study was conducted in one large contiguous plot which was sampled repeatedly, we used a mixed-effects framework to control for possible spatial and temporal autocorrelation in the data. We used ‘clusters’ of lantana as a random effect. A cluster was defined as a group of cells that had the same lantana density in the adjacent or diagonal directions; cells within a cluster were expected to be more spatially correlated than cells belonging to different clusters. In addition, cells within a cluster were expected to change more similarly in time, than cells of a different cluster. Thus, the random effect of ‘clusters’ was expected to account for both spatial as well as temporal autocorrelation in this data set. In order to account for unequal intervals between two consecutive enumerations, we used a ‘lag’ term that was equal to the number of years across which data were not available. The lantana density at a given time step, was thus modelled as a function of per-cell lantana density, heterospecific density and basal area of other woody trees, inter-census rainfall and fire occurrence in the previous time step, a lag term, TWI and a random term for spatial clusters of lantana from the previous time step. The two-way interaction between rainfall and fire and rainfall and TWI were also considered. Instances of fire for a given time step preceded the duration from which rainfall was estimated. These two abiotic factors were thus assumed to be independent of each other and an interaction between these two terms was thus considered appropriate. All variables were scaled by subtracting the mean and dividing by the standard deviation in order to bring them to comparable ranges and to make the interpretation of model coefficients easier. Since we were interested in how lantana density changed in the same location from one time step to another, we selected a random subset of ∼75% of the cells to build the model and used the remaining 25% to test the model. Models were fit using the clmm2 () function of the ordinal package (Christensen 2012) in the statistical programming language R, version 2.15.1 (R Development Core Team 2012). The global model was simplified by stepwise removal of terms that had a *P*≥0.05, starting with the terms that had the highest *P* value. Significance of model terms was assessed by comparing non-simplified and simplified models using the likelihood ratio tests of the anova () function; models were considered to perform equally well at a difference of *P*≥0.05 and simpler models were retained. Predicted densities from the model were used for model verification using the test dataset. A given cell was expected to successfully change to a new density category with a model predicted probability obtained from the predict () function. Model accuracy was checked using a contingency table of observed frequency of occurrence of different lantana densities and those predicted by the model, and computing a gamma statistic of association [32] (Goodman and Kruskal 1954) for the same. Significant interactions were interpreted graphically by back-calculating the probability of obtaining a given density category from the estimated log-odds of getting that density category or lower (Appendix S2).

## Results

**Figure 2:**
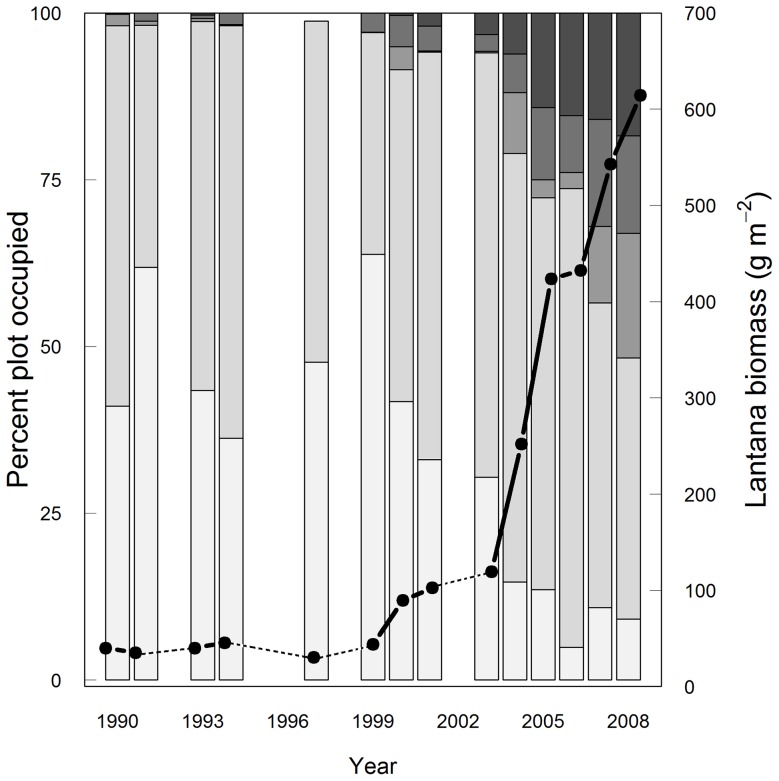
Frequency of occurrence (bars) and biomass (line) of lantana in the MFDP from 1990–2008. Light grey through dark grey represent the increasing density of lantana, i.e, from ‘absent’ through ‘present’, ‘common’ and ‘dense’ to ‘very dense’. The five density categories of lantana occurred with varying frequencies across the time period sampled; the lower density categories occurred less frequently and the higher density categories occurred more frequently with time. Gaps and dotted lines indicate fire years or missing data. The dry weight biomass of lantana is based on a study conducted in the year 2011. Note that for the purpose of analyses, the ‘absent’ and ‘present’ categories were combined to form the low-density ‘present’ category while the ‘common’ and ‘dense’ categories were combined to form a moderate density ‘common’ category.

The frequency of occurrence of higher density categories of lantana increased over the time period of this study, while that of the lower density classes decreased (Fig. 2). The increase in the frequency of occurrence of the ‘very dense’ category from 2% in 2000 to 18% in 2008 was noteworthy, as it indicated rapid intensification of invasion. The frequency of occurrence of the ‘present’ category has been relatively high and constant at 40 to 60% during 1990–2008. Total lantana biomass for the MFDP increased from 40 g m^−2^ in 1990 to 615 g m^−2^ in 2008 (Fig. 2).

### Model Outputs and Parameter Estimates

The random effect accounting for clustering of lantana had a large standard deviation of 2.38, indicating the considerable effect of spatial clumping in this plant. Of all the fixed effects considered in the model, only ground vegetation dropped out of the model as being insignificant. *H. isora* neighbourhood, per cell lantana density, lag, basal area of large trees and the two-way interaction between rainfall and TWI as well as between rainfall and fire were found to significantly affect transitions in lantana density (Table 2). The model had high overall predictability, with a gamma score of 0.78. However, at the level of individual density categories, the model predicted the ‘present’ category the best with an accuracy of 98%, while the only 35% and 40% of the denser categories were accurately predicted. The prediction was the poorest for the ‘common’ category, with 58% of such cells being wrongly predicted as the ‘present’ category.

**Table 2:** Output of the simplified cumulative link mixed model.

		Coef	S.E.	Wald Z	*P*
**Threshold coefficients**	Present to common	−2.59	0.09	−27.91	–
	Common to very dense	−0.02	0.08	−0.25	–
**Fixed effects**	Lantana (Common)	−0.09	0.25	−0.36	0.72
	Lantana (Very Dense)	2.32	0.35	6.52	<0.001
	Lag	−2.74	0.18	−14.84	<0.001
	Rainfall	0.38	0.12	3.19	0002
	Fire frequency	0.48	0.19	2.49	0.01
	Basal area (trees)	0.03	0.02	2.05	0.04
	*Helicteres isora*	−0.06	0.02	−3.44	0.001
	TWI	0.60	0.02	34.10	<0.001
	Rainfall × TWI	0.65	0.28	−2.32	0.02
	Rainfall × Fire frequency	−0.11	0.02	−6.13	<0.001
**Random effect**	**Variance**	**Std. Dev.**			
Spatial cluster	5.65	2.38			

All predictors, corresponding to the time step immediately before that of the current time step, were scaled by subtracting the mean and dividing by the standard deviation in order to make them comparable. The overall predictability of the model was high, with a gamma score of 0.78. TWI – topographic wetness index of a cell, ‘×’ – interactions between factors.

### Biotic Influences on Lantana Invasion

The term for ground vegetation dropped out of the model as being non-significant, indicating that competition from grasses and other invasive species had little or no effect on invasion by lantana. The term for lantana density in a given cell was found to be highly significant (Likelihood Ratio Test, LR statistic  = 52.96, *P*<0.001), indicating that lantana density in the previous time step was an important predictor of lantana density in the next time step. The odds ratio (calculated from the coefficients in Table 2; see Appendix S2 for details) of getting a given lantana density or higher was 0.9 at lantana ‘common’ relative to lantana ‘present’ category, while it was 10.2 at lantana ‘very dense’ relative to lantana ‘present’ category. Thus, cells with dense lantana in the previous time step were likely to remain so or go into a higher density state in the next time step. All else being constant, for a lag of 1 year, the odds ratio of getting a given lantana density category or higher decreased from 15.6 to 15.3 at 2 SD below and above the mean *H. isora* stem density, respectively. Thus, the probability of getting a higher lantana density class decreased with increasing *H. isora* stem density. However, since the rate of change of odds is low, this also indicates that lantana persisted at low densities (‘present’ category) across all densities of the competing shrub. On the other hand, for the same time lag, the odds ratio of getting a given lantana density category or higher, increased from 14.9 to 16.4 at 2 SD below and above the mean basal area of trees, indicating that lantana invasion was positively associated with basal area.

### Abiotic Influences on Lantana Invasion

**Figure 3:**
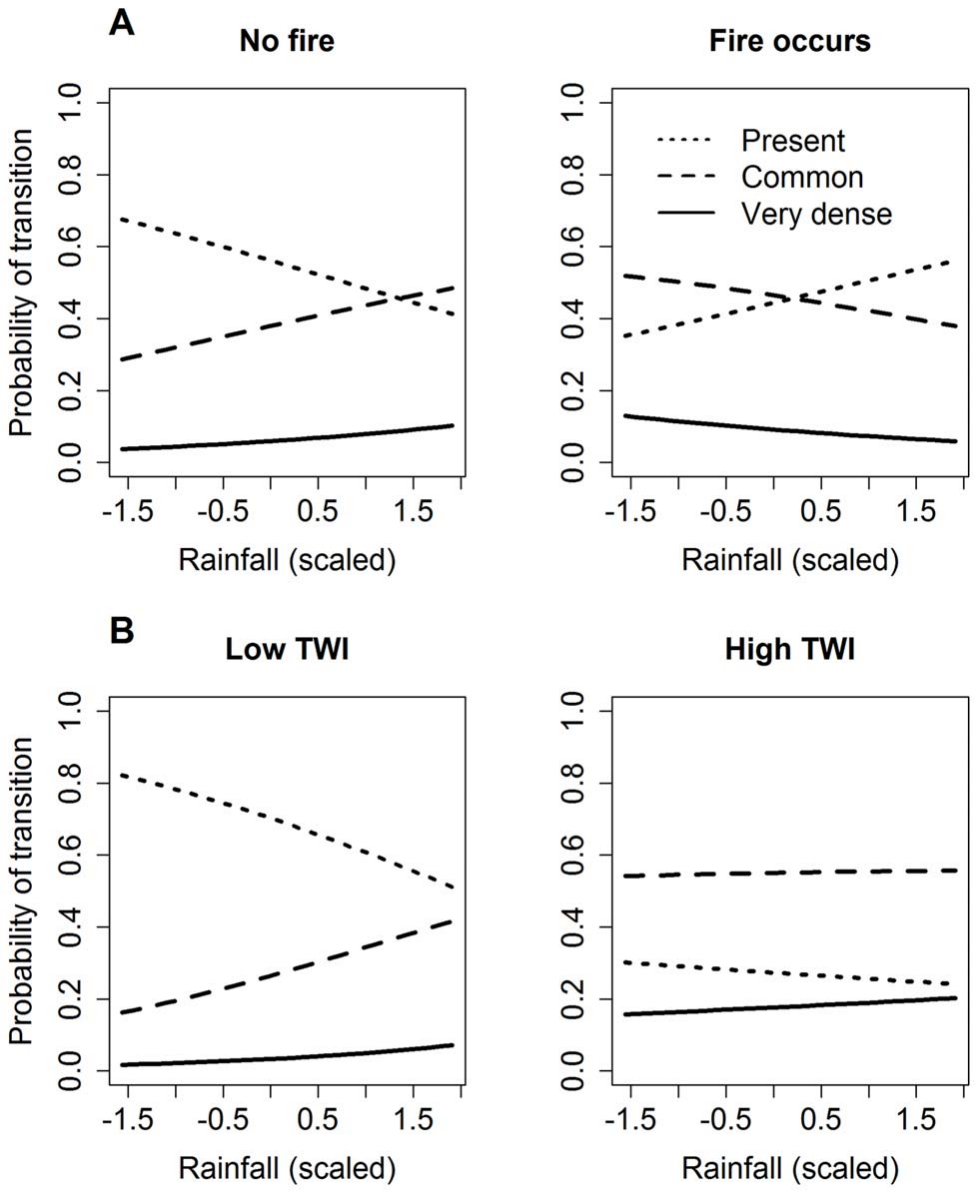
Model predictions for transition from lantana ‘present’ state to higher density categories. Over a lag of 1-fire interaction (a) and rainfall-topographic wetness index interaction (b). See text for details. Note that the x-axis (annual rainfall) is scaled by subtracting the mean and dividing by the standard deviation, thus a value of ∼0 indicates the mean rainfall, values <0 indicate below-average rainfall and vales >0 indicate above-average rainfall. The limits of the x axes are the 95% quantiles of the scaled variable.

The interaction between rainfall and fire was graphically explored by computing the probability of lantana ‘present’ category remaining so, or changing into a higher density category while holding all other predictors constant. In the absence of fire, for a lag of 1 year, the probability of lantana ‘present’ remaining so decreased with increasing rainfall, but that of lantana going to a higher density category increased (Fig 3a). When a single fire event occurred in the interim, lantana became denser at lower rainfall values (Fig 3a). The interaction between rainfall and TWI was also a significant predictor of lantana density (Table 2). All else being constant, in drier parts of the plot (low TWI), the probability of the ‘present’ category remaining so decreased and that of higher density categories increased with increasing rainfall. In wetter parts of the plot (high TWI), however, the probability of finding the ‘common’ category was always higher than finding the ‘present’ category and that of the ‘very dense’ category increased with increasing rainfall (Fig 3b).

## Discussion

This study examines the influences of biotic and abiotic factors on invasion by lantana in a seasonally dry tropical forest. Our observations indicate that over a span of 18 years, the transitions in lantana density have been strongly in favour of the higher density categories in the MFDP.

### Biotic Influences on Lantana Invasion

The overall spread and biomass of lantana in the MFDP increased considerably during the latter half of this study (Fig. 2). Lantana in the MFDP is not self-limiting yet, as lantana ‘common’ cells tended to remain so or go into a higher density state in the next time step. The emergence of higher density categories, however, may be largely due to an increasing clonal population of lantana and not necessarily the emergence of new individuals. In a common garden experiment we found the germination success of lantana to be only 12% under ambient shade conditions (G. R., unpublished data). Even though low germination rates could be offset by high propagule pressure – up to 1.2 million seeds per hectare [33] – increasing lantana density in the same cell is most likely due to its coppicing ability on being top-killed (due to fire or mechanical injury) or due to vegetative propagation [34].

As expected, lower lantana density was associated with higher stem density of the competing native woody shrub *H. isora*. This is also borne out by the fact that both lantana and *H. isora* seem to have a preference for similar habitats in the MFDP (near streams). *H. isora* stem densities have been observed to increase since 2005, about 2–3 years following the expansion of lantana in the plot. Although the competitive effects of increasing stem densities of *H. isora* are likely to prevent intensification of lantana invasion, the persistence of lantana in low densities may result in further invasion in the future. Contrary to expectation, higher basal area of trees was associated with higher lantana density. At the landscape level, lantana density has been found to be negatively correlated with tree density in another dry forest of southern India [35]. We speculate that owing to the MFDP being a relatively open forest (∼250 stems of >10 cm dbh per ha), it is likely that even at the high values of basal area in a cell, there is sufficient light reaching the ground to support the persistence of dense lantana.

Native grasses and exotic *C. odorata* were found to have no influence on lantana invasion although grasses themselves may be directly affected by the presence of lantana. In an independent study spanning 2 years (2008–2010) we observed that the grass cover under dense lantana was consistently low across seasons, covering an average of 34.4% sampled points outside lantana and only about 7.1% sampled points under dense lantana (Figure S3 in Appendix S3). Communities dominated by exotic species are often known to be replaced by other exotics over annual and decadal time scales [36]. We observed an increase in the ‘common’ and ‘dense’ categories of *C. odorata* occupying 40% and 31%, respectively, of all cells up to the year 2004, followed by a decrease in frequency of occurrence the following year. This decrease coincides with an increase in the frequency of occurrence of higher density classes of lantana in the MFDP.

### Abiotic Influences on Lantana Invasion

Melbourne et al (2007) highlight the necessity of having both spatial and temporal variations in the environment for an invasive species to be successful. Invasive species could persist at low numbers for long times and then suddenly increase in their population by the opportunistic use of available resources [10]. It has also been hypothesised that invasive plants have high resource use efficiency, enabling high performance even in resource-poor conditions [25]. Alternatively, we find that lantana could also be opportunistically compensating for resources that are spatially unavailable but are variably abundant in time. High rainfall was found to be beneficial for lantana in the drier parts of the plot while, as expected, wetter parts of the plot always supported high densities of lantana. Increase in the previous-season's rain has been found to favour the intensification of invasion [13], a result that is supported by our study.

Changes in disturbance regimes may result in the expansion of the ranges of invasive species [37]. The MFDP has experienced considerable changes in both fire and rainfall patterns over the timeframe examined (Fig. 1). Fire has been shown to intensify invasions in certain ecosystems [22], [38], although there is evidence that this is not true in all cases [39]. Contrary to what has been reported from earlier studies [40], we found that fire did not directly promote lantana invasion, at least in the short term. However, fire interacted with temporal availability of moisture. We found that in the absence of fire, transitions to higher density were more likely with increasing rainfall. When fire occurred, the transitions to higher density occurred at lower than average annual rainfall while the trend reversed at higher values of rainfall. This trend indicates that the occurrence of a disturbance event facilitated the spread of this invasive even though the availability of another resource (moisture) was reduced.

The exponential phase of lantana expansion (Fig. 3) occurred during 2002–2004 after an intense fire (in early 2002 that itself followed the onset of drought beginning 2000) followed by two drought years (2002–2003). The patterns of increase in lantana density observed in the MFDP are concurrent with the patterns observed in the dry deciduous forest of Biligiri Rangan Hills in southern India, where the mean stem density of lantana increased 10-fold in 11 years [41]. Indeed, during the decade of 2001–2010, a significant expansion of lantana also occurred over a large area in the southern Western Ghats of India, including Nagarahole, Bandipur and other parts of Mudumalai, thereby pointing to the influence of a set of common environmental factors that operated at a regional scale. The drought phase during 2000–2003 was common to all these sites and lantana may have eventually benefitted from such conditions through an interaction with fire. We thus speculate that fires coupled with droughts promote lantana invasion and that future changes in fire regimes (increasing fire frequency) and rainfall patterns (increasing number of droughts) may result in further expansion of this shrub.

### Management Implications

The effects of lantana on native species have been found to be variable [41], [42]. Although lantana is currently expanding its range and a large proportion of the tropics are suitable for further expansion [43], predicted ranges are likely to decrease under future climate change [44]. Worldwide, diverse control measures (physical, chemical and biological) have been largely unsuccessful in effectively curtailing the spread of lantana [43]. Traditional control methods often involve the removal of above-ground lantana biomass or burning which are temporarily effective, but result in profuse coppicing post-rain; uprooting lantana or damaging its coppicing meristem is a more effective control measure [33]. Understanding the varying environmental factors that influence the process of invasion in different ways may aid managers to determine suitable times of the year, season or phase of invasion that could be potentially managed. High-risk zones such as moister stream-sides and high risk years such as those in which droughts and fires occur simultaneously could be identified and management activities focussed accordingly.

## Supporting Information

Appendix S1 **Details of the study site that could not be included in the main text.** Figure S1.1 gives the change in lantana density states from 1990 to 2008 in the MFDP. Figure S1.2 gives the inverse relationship between monthly rainfall and fire occurrence.
(DOC)

Appendix S2 **Description of the method used to obtain odds ratios from a cumulative link mixed effects model.**
(DOC)

Appendix S3 **Details of a study that was conducted to understand the effects of dense lantana on the ground vegetation in the MFDP.** Figure S3 gives a comparison of the proportion area occupied by grasses under and outside dense lantana over a period of two years.
(DOC)
