# Supplementary material for: Long-Term Environmental Correlates of Invasion by Lantana camara (Verbenaceae) in a Seasonally Dry Tropical Forest
Source: PLoS One. 2013 Oct 22;8(10):e76995. doi: 10.1371/journal.pone.0076995 (PMC3805544; doi:10.1371/journal.pone.0076995)
Supplement: Appendix S1 — Details of the study site that could not be included in the main text. Figure S1.1 gives the change in lantana density states from 1990 to 2008 in the MFDP. Figure S1.2 gives the inverse relationship between monthly rainfall and fire occurrence. (DOC) [file pone.0076995.s001.doc]

# Supporting Information

## Appendix S1 - Study site details

Density maps of invasive *Lantana camara* at the Mudumalai Forest Dynamics Plot

**
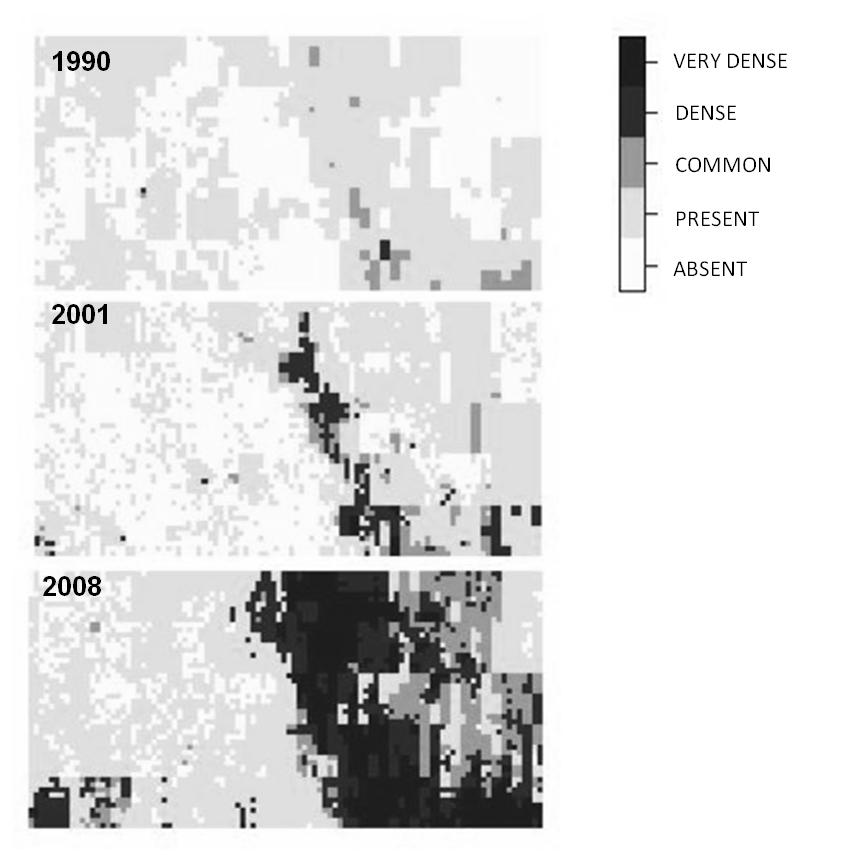
**

**Figure S1.1** Spatial maps of the qualitative density categories of lantana – ‘absent’, ‘present’, ‘common’. ‘dense’ and ‘very dense’ – in the MFDP for the years 1990, 2001 and 2008. Pixels represent 10m × 10m plots

Co-varying environmental factors at the MFDP


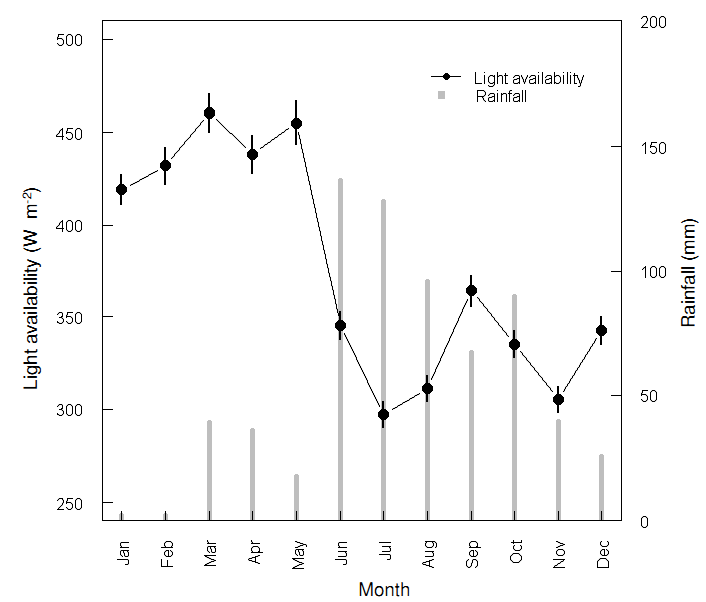


**Figure S1.2** The inverse relationship between solar insolation and monthly rainfall at the MFDP. The solid circles and line represents variation in mean (± SE) light availability across all months of the year for the years 2005-2008, while the grey bars represent total monthly rainfall (mm).
