# Supplementary material for: Long-Term Environmental Correlates of Invasion by Lantana camara (Verbenaceae) in a Seasonally Dry Tropical Forest
Source: PLoS One. 2013 Oct 22;8(10):e76995. doi: 10.1371/journal.pone.0076995 (PMC3805544; doi:10.1371/journal.pone.0076995)
Supplement: Appendix S2 — Description of the method used to obtain odds ratios from a cumulative link mixed effects model. (DOC) [file pone.0076995.s002.doc]

# Supporting Information

## Appendix S2 - Inferring odds ratios and probabilities from a cumulative link mixed model

Note: Equations have been adapted from accessory information available on the ‘ordinal’ package (Christensen, R. H. B., 2012, “A Tutorial on ﬁtting Cumulative Link Mixed Model with clmm2 from the ordinal Package”, URL: http://cran.r-project.org/web/packages/ordinal/vignettes/clmm2 tutorial.pdf)

### S2.1 The cumulative link mixed model

In a cumulative link mixed model, when the response has *j* ordinal levels, the cumulative probability that a given observation is less than the *j*th level is:

logit(*P*(Y ≤ *j*)) = *θ_j_*  – *β_1_*x_1_ – *β_2_*x_2_ – ... *β_n_*x_n_  – *u*R

where:

*θ_j_* is a threshold coefficient (or intercept), indicating the cut-off point at which a lower ordinal level changes to the next higher level

*β_1_, β_2,.._. β_n_* are the coefficients of the predictors x_1_, x_2_,...x_n_

R is a random effect and the distribution *u*R corresponds to a normal distribution with mean 0 and standard deviation σ^2^

In the current study *j* corresponds to one of 3 values: present, common and very dense (increasing order of biomass). Per-cell probability of getting a given lantana density or less (at current time step *T*) was thus as follows for the 3 different starting densities (at previous time step *T – 1*)

1. When lantana was ‘present’ at *T-1*

logit(*P*(Y_T_ ≤ *j*)) =  *θ_j_*  *–* (*–* 2.74)*L *–* (*–* 0.06)*H_T-1_ *–*  0.03*B _T-1_ *–* 0.38*R _T-1_  *–* 0.48*F _T-1_ *–* 0.6*TWI _T-1_ *–* (*–* 0.65)* R _T-1_* F _T-1_ *–* (*–*0.11)* R _T-1_*TWI _T-1_

1. When lantana was ‘common’ at *T-1*

logit(*P*(Y_T_ ≤ *j*)) =  *θ_j_*  *–* (*–*0.09)*–* (*–* 2.74)*L *–* (*–* 0.06)*H_T-1_ *–*  0.03*B _T-1_ *–* 0.38*R _T-1_  *–* 0.48*F _T-1_ *–* 0.6*TWI _T-1_ *–* (*–* 0.65)* R _T-1_* F _T-1_ *–* (*–*0.11)* R _T-1_*TWI _T-1_

1. When lantana was ‘very dense’ at *T-1*

logit(*P*(Y_T_ ≤ *j*)) =  *θ_j_*  *–* 2.32 *–* (*–* 2.74)*L *–* (*–* 0.06)*H_T-1_ *–*  0.03*B _T-1_ *–* 0.38*R _T-1_  *–* 0.48*F _T-1_ *–* 0.6*TWI *–* (*–* 0.65)* R _T-1_* F _T-1_ *–* (*–*0.11)* R _T-1_*TWI

where:

*θ_j_* = threshold coefficient for transition into the *j*th density category at time *T*

L = lag term (1,2 or 3) for number of gap years between two consecutive enumerations

H_T-1_ = *Helicteres isora* stem density at time *T-1*

B _T-1_ = Basal area of trees at time *T-1*

R _T-1_ = Annual rainfall received at time *T-1*; average rainfall if L > 1

F _T-1_ = cumulative number of fire events (0,1,2 or 3) between *T* and *T-1*; F _T-1_ > 1 only when L > 2

TWI = topographic wetness index of the cell

For examining the effects of a single predictor, all others were held at a mean (scaled) value of zero. When an interaction was explored, all predictors except those interacting were set to zero. In this paper, only results from eqn. a. have been reported.

### S2.2 Calculating the odds ratio

The odds ratio for getting an observation that is ≥ *j* for a treatment (categorical variable) is given as exp(*β_treatment_*). In this study, the only categorical variable is lantana density in the previous time step. The *β*s for Lantana (Common) and Lantana (Very Dense) in Table 2 (-0.09 and 2.32 respectively) give the log odds of getting a lower lantana density v/s a higher lantana density in the current time step, when the cell was occupied by ‘common’ and ‘very dense’ categories relative to the ‘present’ category of lantana in the previous time step. Thus the odds ratio for getting a lower (present or common) v/s higher density (common or very dense) of lantana in the current time step when lantana was ‘common’ relative to the ‘present’ category in the previous time step =

exp(-0.09) = 0.92

but at lantana ‘very dense’ relative to the ‘present’ category, the odds ratio is:

exp(2.32) = 10.17

### S2.3 Calculating the probability of getting a single density level

Probabilities for different density categories for a given predictor ‘x’ and time lag ‘L’ were calculated as follows:

*P*(‘present’) = logit^-1^(*θ*_present to common_ – *β*x – *β_lag_*L)

*P*(‘common’) = logit^-1^(*θ*_common to very dense_ – *β*x – *β_lag_*L) – logit^-1^(*θ*_present to common_ – *β*x – *β_lag_*L)

*P*(‘very dense’) = logit^-1^ (1 – (*θ*_common to very dense_ – *β*x – *β_lag_*L))

Such that, *P*(‘present’) + *P*(‘common’) + *P*(‘very dense’) = 1

Where ‘x’ is a vector of values, limited at the 95% quantile values of the scaled predictor from the observed data, while ‘L’ was held constant at 1 such that probabilities could be calculated at an average time lag of 1 year. In case of the rainfall-fire and rainfall-TWI interaction, the coefficient of the interaction term was subtracted from the threshold coefficients as well, before computing the inverse logit. The inverse logit was computed using the plogis() function in R (version 15.1).
