# Supplementary material for: Long-Term Environmental Correlates of Invasion by Lantana camara (Verbenaceae) in a Seasonally Dry Tropical Forest
Source: PLoS One. 2013 Oct 22;8(10):e76995. doi: 10.1371/journal.pone.0076995 (PMC3805544; doi:10.1371/journal.pone.0076995)
Supplement: Appendix S3 — Details of a study that was conducted to understand the effects of dense lantana on the ground vegetation in the MFDP. Figure S3 gives a comparison of the proportion area occupied by grasses under and outside dense lantana over a period of two years. (DOC) [file pone.0076995.s003.doc]

# Supporting Information

## Appendix S3- Enumeration of ground vegetation under and outside dense lantana

The thicket forming habit of lantana was expected to reduce light availability at ground level. This was in turn expected to affect the plants growing at ground level. The effect of shading by lantana was also expected to vary with season. To understand how lantana affects grass species (*Themeda cymbaria*, *Imperata cylendrica* and *Cymbopogon flexuosus*) that form a large proportion of the ground vegetation at the Mudumalai Forest Dynamics Plot, observations on the same were made under and outside dense lantana thickets

This study was an extension of the study on the effects of lantana on native species’ seedlings (Ramaswami and Sukumar 2013). A total of 40 10m × 10m plots (stratified on the basis of lantana density) were monitored from June 2008 to February 2010 at bimonthly intervals. 20 plots belonged to the lantana ‘absent’ category, while the remaining 20 belonged to the lantana ‘very dense’ category, henceforth referred to as LA and LD plots respectively. Within each plot, 5 point measurements on each side were made at ~ 1m from the boundary. A total of 20 point measurements per plot were thus taken. At each point, the presence/absence of grasses was noted. At the end of each enumeration, the total number of points occupied by grasses within all plots was converted to a percentage value. Inferences were made assuming that the point samples represented the overall distribution of grasses.

Across the duration of the study, grass species seemed to be affected by the presence of dense lantana. On an average, grasses covered 34.4% of sampled points in LA (lantana absent) plots and only 7.1% of sampled points in LD (lantana dense) plots. It was inferred that dense lantana may be detrimental to the persistence of grasses.


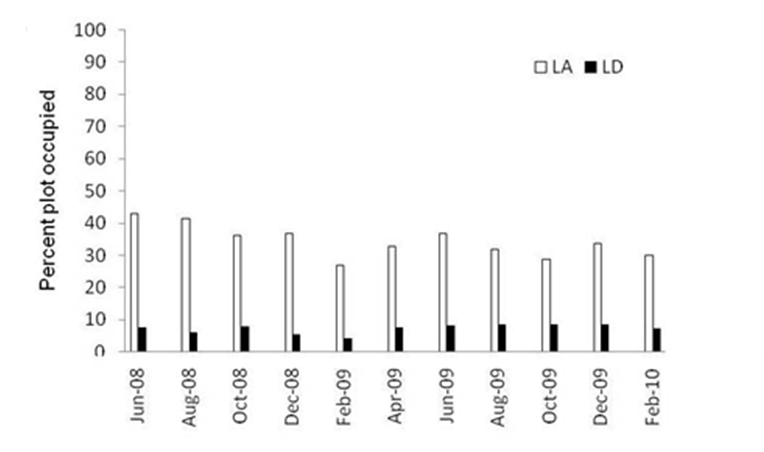


**Figure S3** Percentage of sampled points occupied by grasses was consistently higher in lantana ‘absent’ (LA) than lantana ‘very dense’ (LD) plots between June 2008 and February 2010
